# The Post-Apoptotic Fate of RNAs Identified Through High-Throughput Sequencing of Human Hair

**DOI:** 10.1371/journal.pone.0027603

**Published:** 2011-11-16

**Authors:** Gloria K. Lefkowitz, Anandaroop Mukhopadhyay, Christopher Cowing-Zitron, Benjamin D. Yu

**Affiliations:** Stem Cell Program, Division of Dermatology, Department of Medicine, Institute for Genomic Medicine, University of California San Diego, San Diego, California, United States of America; University Hospital Hamburg-Eppendorf, Germany

## Abstract

The hair of all mammals consists of terminally differentiated cells that undergo a specialized form of apoptosis called cornification. While DNA is destroyed during cornification, the extent to which RNA is lost is unknown. Here we find that multiple types of RNA are incompletely degraded after hair shaft formation in both mouse and human. Notably, mRNAs and short regulatory microRNAs (miRNAs) are stable in the hair as far as 10 cm from the scalp. To better characterize the post-apoptotic RNAs that escape degradation in the hair, we performed sequencing (RNA-seq) on RNA isolated from hair shafts pooled from several individuals. This hair shaft RNA library, which encompasses different hair types, genders, and populations, revealed 7,193 mRNAs, 449 miRNAs and thousands of unannotated transcripts that remain in the post-apoptotic hair. A comparison of the hair shaft RNA library to that of viable keratinocytes revealed surprisingly similar patterns of gene coverage and indicates that degradation of RNA is highly inefficient during apoptosis of hair lineages. The generation of a hair shaft RNA library could be used as months of accumulated transcriptional history useful for retrospective detection of disease, drug response and environmental exposure.

## Introduction

Apoptosis is a cellular program utilized by multicellular organisms to eliminate cells during development or in surveillance of foreign or abnormal cells altered by viral infection or neoplastic transformation [1], [2]. Cells undergoing apoptosis undergo nuclear, chromatin, and additional organelle changes induced by a cascade of molecular signals involving several proteases and deoxyribonucleases [3]. Defects in programmed cell death results in the abnormal accumulation of cells, altered morphogenesis during development, and the persistence of transformed cells in disease.

The hair follicle is a model of apoptosis [4], [5]. Programmed cell death in the hair follicle occurs both during its normal growth and differentiation and during an involutional stage called catagen. Ultrastructural studies demonstrate that hair follicle cells undergo a specialized form of apoptosis called cornification during terminal differentiation [6]. During this process, the nuclear membrane is lost, and the chromatin becomes less coarse. In addition, evidence of DNA damage as detected by direct end-labeling of nicked DNA and indirect double-strand break activity have been identified during early and late stages of hair differentiation [4], [7], [8]. Skin-specific endonucleases such as DNase1L2 target genomic DNA during cornification [3], [9], and in the absence of *DNase1L2*, nuclear DNA persists in the hair and causes hair fragility [10]. The external hair is defined as the hair shaft and consists largely of the proteinaceous remnants of three cell types [11]. The three hair shaft cell types, the outer cuticle, cortex and central medulla, originate from a self-renewing progenitor population called the matrix [12]. The hair matrix also produces supportive, non-hair shaft cell types, which form a rigid sheath around the hair shaft and enable the hair to exit through the skin. Much of the strength of the hair shaft and sheath come from intermediate filament proteins called keratins and keratin-associated proteins (KRTAPs) which become crosslinked by several enzymes during terminal differentiation [13], [14].

While DNA degradation is a common hallmark of apoptosis, the targeting of RNA for degradation during apoptosis is unclear. The removal of RNA following apoptosis may be of importance as released endogenous RNA appears to activate inflammation and the innate immune response [15]. In viable cells, multiple mechanisms exist to regulate the homeostasis of mRNAs, including nonsense-mediated decay, targeting of AU-rich elements, microRNA-mediated destabilization, and others [16], [17]. During some forms of apoptosis, RNases with broad specificity are upregulated such as IFN-gamma induced RNase L [18]. In addition, specific endonucleolytic activities, which target 28S ribosomal RNA, have been observed during apoptosis and appear to trigger independently of DNA degradation [19], [20]. More recently, an endonuclease RNase III, DICER, has been shown to shift specificities between RNA to DNA during apoptosis [21]. These several observations indicate that during apoptosis, RNA and DNA targeting are distinctly regulated but the final fate of RNAs following apoptosis is unknown.

Here we investigate the extent to which RNAs survive apoptosis by studying the external hair shaft of mice and humans. We hypothesized that pools of small RNAs, in particular, regulatory microRNAs (miRNAs), might preferentially survive destruction by nucleases during the process of cornification, because of their inherent stability [22]. Using RNA sequencing and real-time quantitative PCR, we instead find that many types of RNAs survive apoptosis including miRNAs and mRNAs and persist long after hair shaft formation at several centimeters from the scalp. The post-apoptotic human hair contains the thousands of mRNAs, miRNAs, and other small RNAs and accurately reflects tissue of origin. Finally, we find that the RNA of the post-apoptotic hair shares similar patterns of intragenic coverage as RNA from viable keratinocytes. Hence, the removal and destruction of RNA post-cornification appears to be highly inefficient in the keratinized structure of the hair and potentially provides an ideal tissue for studies of genetic and acquired diseases.

## Results

### Detection of lineage-specific miRNA and mRNA in mouse hair shafts

**Figure 1:**
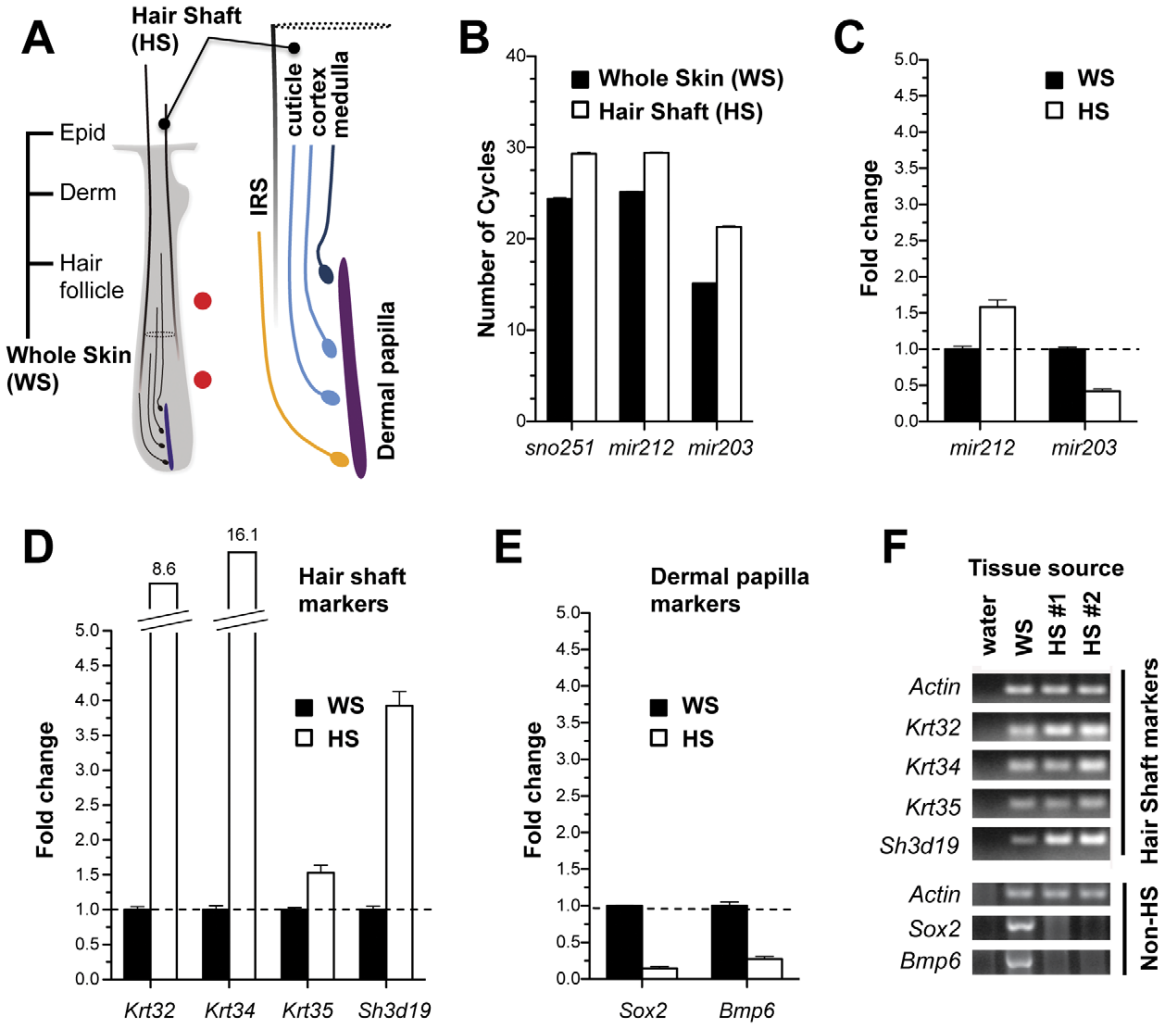
Detection of tissue-specific miRNA and mRNA from mouse hair shafts. (A) Schematic diagram detailing source of tissue used in mouse studies. Hair shaft (HS) refers to the external portion of hair used for RNA isolation. Whole skin (WS) encompasses the epidermis, dermis, and the entire hair follicle, which includes cells fated to become the hair shaft (blue), supporting non-hair shaft cell populations (inner root sheath, orange and dermal papilla, purple). Red circles represent the earliest and latest regions of detected apoptosis. (B) Differential expression of snoRNAs and miRNAs in whole skin versus external hair shaft from 2–3 week old mice. Detection levels are displayed in cycle numbers normalized for equal input total RNA. Higher cycle numbers indicate lower levels of detection. (C) Mouse *mir-212* and *mir-203* levels normalized to *snoRNA-251* levels reveal increased *mir-212* and greater than two-fold reduction of *mir-203* in mouse hair shaft relative to whole skin. (D) Detection and quantification of cuticle, cortex and medulla-specific transcripts in hair shaft vs. whole skin reveal patterns of enrichment. (E) Assay for dermal papilla-specific transcripts in hair shaft vs. whole skin total RNA. (F) Gel electrophoresis of amplification products of hair shaft and non-hair shaft (non-HS) genes in whole skin and hair shaft.

In mice, the transcriptional profiles of living hair follicle-specific miRNAs and mRNAs have been previously examined [23], [24]. Based on these expression profiles, we examined whether miRNAs and mRNAs specifically expressed in the hair follicle could also be detected in the external hair shaft and tested whether non-hair shaft transcripts were excluded. As comparison, we utilized whole mouse skin, which contains all lineages of the hair follicle, epidermis, dermis and adipose tissue (Fig. 1A). Using quantitative real-time PCR, miRNAs were readily detected in the hair shaft at 3–4 cycles higher than from equivalent amounts of whole skin RNA (Fig. 1B). Relative to control RNAs, e.g. *snoRNA-251*, we found evidence for lineage specificity using miRNA expression (Fig. 1C). Significantly, the level of a miRNA (*mir-203*), which is considered to be epidermal-specific [25], was ten fold less abundant in the mouse hair shaft compared to whole skin. These findings indicate that miRNAs are sufficiently intact in the hair shaft for specific detection by quantitative real-time PCR (qRT-PCR).

As hair keratins are abundantly expressed during hair development, we next investigated whether their transcripts can be detected in the external hair shaft. In addition, hair keratin genes are also expressed by specific cell types and identify the cell type of origin for RNAs extracted from external hair (Fig. 1D). Several lineage-specific keratin [13] and central medulla genes [26] could be detected in the post-cornification hair shaft (Fig. 1D, F). Relative to whole skin, *Keratin 32* (*Krt32*), *Krt34*, and *Krt35* were detected at 8.6, 16.1 and 1.5-fold higher in the external hair. *Sh3d19*, a medulla-specific gene, was also relatively more abundant in the hair shaft. The enrichment of these RNAs in hair shaft relative to whole skin occurs as hair follicle transcripts are diluted by other tissues present in whole skin. As a negative control, genes expressed by tissues that do not contribute cells to the hair shaft were examined. One such tissue, called the dermal papilla, is a closely associated mesenchymal population that supports hair growth but does not itself contribute cells to the growing hair shaft. We found that two dermal papilla-specific genes, *Sox2* [27] and *Bmp6* [28], were significantly reduced in expression in the hair shaft, only detectable at 6.8 and 3.7 fold levels below whole skin, respectively (Fig. 1E, F). In sum, these studies indicate that miRNAs and mRNAs of at least three cell types persist in the external hair of mice contains both miRNAs and mRNAs and that cornification does not sufficiently destroy these RNAs to prevent their detection.

### Generation of A Human Hair RNA-Seq Library

**Figure 2:**
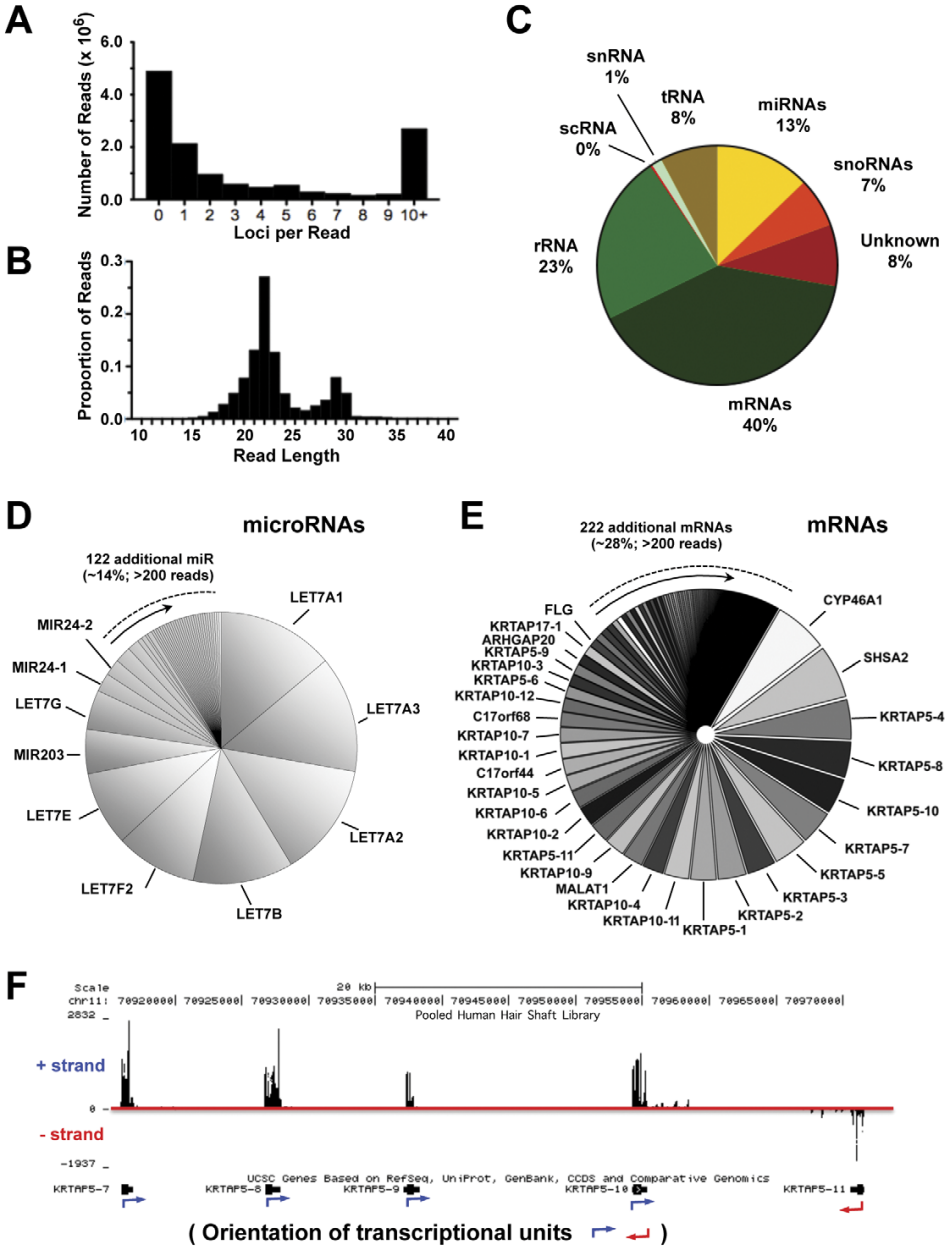
Generation of a human hair shaft small RNA library. Small RNA library constructed from RNA pooled from hair shafts of five individuals. (A) Uniqueness of RNA sequences. A large portion of reads can be mapped to 1 or more loci in the human genome (hg18). (B) Distribution of trimmed read lengths demonstrates two peaks at 22 and 29 nucleotides. (C) Types of RNA retained in the human hair shaft. Percentages of genomic aligned RNAs map to known mRNAs, miRNAs, rRNAs, unannotated RNAs, and others. (D) Complexity of miRNA in chart reveals high proportion of *LET7* family members. Only miRNAs with 200 or more hits are demonstrated in chart. (E) Complexity of mRNAs in human hair shaft demonstrated in pie chart. (F) Read coverage of hair shaft RNA library of a 60 kb genomic window, representing of a portion of the KRTAP5 gene cluster. Alignment of reads demonstrates enrichment of sequences to transcriptional units and strand preservation of known transcript orientation of *KRTAP5-7*, *5-8*, *5-9*, *5-10*, and *5-11* genes. These findings confirm absence of contaminating genomic DNA sequences.

We considered the possibility that residual RNAs in the mouse hair might reflect its rapid growth and potential persistence of cells rather than the low ribonuclease activity during and after cornification. In humans, hair does not contain viable cells [11] and undergoes 1–2 months of maturation prior to its emergence from the scalp, compared to 1 week in mice [29]. While human hair may be a better model to study post-terminal differentiation hair, studies of human hair versus mouse hair posed unique challenges. First, expression data of miRNAs in the living human hair follicle was limited, and second, gene expression biases may arise from individual variation in gene expression or detection biases due to nucleotide polymorphisms. We addressed these limitations by utilizing parallel sequencing of small RNAs [30] isolated from human hair (Fig. 2). To generate a comprehensive library of RNAs in human hair and to account for some aspects of human variation, we pooled RNA from the hair shafts of five individuals, who varied in gender, hair shape, and origin (demographics detailed in Supplemental Methods). Total RNA isolated from external human hair shafts by this method was ligated to adaptors designed for small RNA reads, reverse transcribed, amplified, size selected and analyzed by Illumina/Solexa-based small RNA sequencing.

From this approach, 13.5 million high-quality reads and 1.2 million unique sequences were obtained. Subsequently, the reads were aligned to the human genome (hg18) and to dedicated small RNA libraries (miRBase and snoBase). A large portion of reads aligned to more than 10 loci or no loci, due to low complexity of sequence, highly repetitive targets or the presence of non-human RNA (Fig. 2A). We also found a high frequency of aligned reads measuring 22 nucleotides, the size of mature miRNAs (Fig. 2B). 7,193 mRNAs were detected with read coverage of 10 or more, and 251 mRNAs were found at 200 or more reads. For alignments that had 200 or more reads, we also identified 449 distinct miRNAs and 339 snoRNAs in the hair shaft library. Percentages of reads of different classes of RNA identified from human genome annotation are shown in Figure 2C.

The vast majority of miRNAs detected in the hair shaft belonged to the *LET-7* family, which plays a role in regulation of proliferation, differentiation, and stem cell maintenance [31] (Fig. 2D, Table S1). The remaining 123 miRNAs account for 21.4% of all miRNAs in the hair shaft. Like the mouse hair, the human hair shaft contained numerous mRNAs encoding genes of the keratin associated proteins (KRTAP) or keratins (Fig. 2E, Table S1). Members of the *KRTAP* family comprise 58.3% of all mRNAs in the hair shaft. Like keratins, *KRTAP* genes function as support proteins, which become highly crosslinked and participate in forming the shape of the hair shaft [32]. Many characteristics of the small RNA library generated from hair allowed us to verify that the sequences originated from RNA rather than contaminating genomic DNA. First, sequence data showed significantly greater coverage of transcriptional units compared to non-transcribed genomic regions (Fig. 2F). Second, the directional nature of the small RNA library preparation revealed specific alignment of sequence reads to the known transcriptional orientation of individual genes.

**Figure 3:**
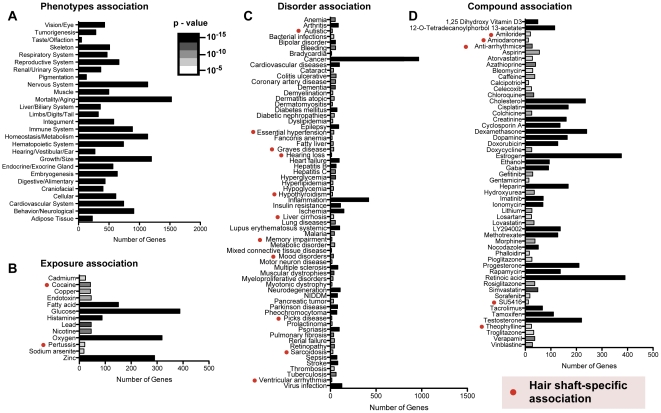
Gene ontology analysis of hair shaft RNA library. (A) Phenotypic association of genes expressed in the hair shaft library. Shown are numbers of genes present in the hair shaft that are associated with organ phenotypes identified by mouse mutations. (B) Environmental exposures pathways associated with genes identified in hair shaft library. Numbers of genes associated with selected exposure pathways are shown. (C) Disorder pathways associated with genes present in hair shaft library. (D) Compound pathways associated with hair shaft RNA library. Gray shading of column bars in all panels indicates lower limits of p-values calculated based on input hair shaft library genes vs. random background genes. Red solid circles denote unique pathway gene sets identified in hair shaft but not keratinocytes.

Many of the mRNAs found in the hair shaft were not specific to the hair follicle. Several gene annotation searches, including a Set-Distiller batch tool [33], identified strong statistical associations to phenotypes and pathways affecting other organ systems (Fig. 3A). 377 pathways involved in environmental and pharmacologic signals and 391 pathways related to specific human disorders were associated with genes detectable in the hair shaft. Given the large 7,193-gene set used to represent the hair shaft, we attempted to address the specificity of non-hair dataset associations by comparing these results to the comparison of an 11,027-gene set representing normal human epidermal keratinocytes (NHEK). The NHEK dataset was chosen, because it still represents epithelial tissue but is also significantly different from the hair shaft in origin and grown under different conditions. Eighteen signatures (p = 3.7×10^−6^ to 1.0×10^−11^) belonging to exposure, disease or medication-induced gene expression showed detection in hair shaft RNA but not NHEK RNA (Fig. 3B–D). These findings suggest that many associations between hair shaft RNAs and non-hair related pathways exist irrespective of the large input gene set. Moreover, these annotation studies suggest that hair shaft tissue may be of significant medical value in the screening of transcribed biomarkers shared with epidermal keratinocytes or uniquely as a source for conditions such as autism, hypertension, and thyroid diseases. Lastly, many of the transcripts present in the hair shaft are themselves genetic targets in human disease (Table S2) or contribute to pathways in involved in response to chemical compounds and drugs (Table S3). The gene ontology characterization of hair shaft RNA profiles provides guidance for the use of the hair shaft to study organismal responsiveness to medications or environmental exposures to chemicals or infections.

### Relative Similarities in RNA content of Viable Keratinocytes and External Hair

**Figure 4:**
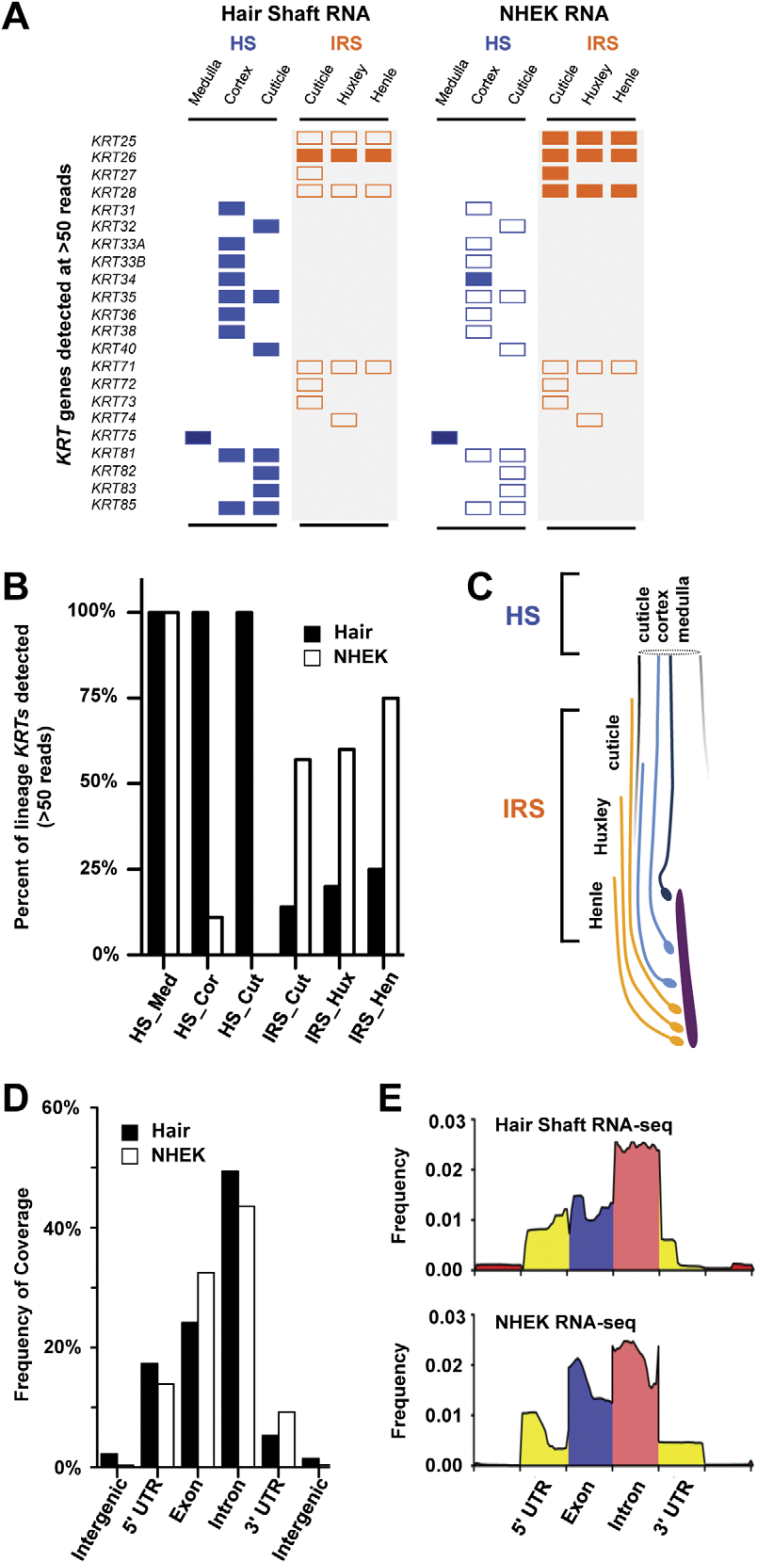
Comparison of hair shaft vs. cultured human keratinocyte RNA sequenced libraries. (A) Cell-type specific detection of human hair keratins (*KRT*) in hair shaft versus normal human epidermal keratinocytes (NHEK). Pattern of KRT expression in six different hair shaft (HS) and non-hair shaft inner root sheath (IRS) are shown in boxes, where filled boxes indicate ≥50 reads detected. (B) Percent of cell-type specific keratin genes detected reveals degree of enrichment of each hair shaft and non-hair shaft compartment in hair shaft vs. NHEK RNA libraries. (C) Illustration demonstrating the spatial distribution of cell types represented by lineage-specific *KRT*, including three hair shaft cell types (blue) and three IRS cell types (orange). (D) Genome-wide comparison of intragenic region coverage by hair shaft vs. NHEK RNA libraries reveals similar representation of most intragenic regions. Exonic coverage was greater in viable NHEK compared to hair, while intronic coverage was more extensive in hair. (E) Distribution of sequence coverage within regions of each domain, e.g. 5′ intergenic, 5′-UTR, introns, exons, 3′-UTR and 3′ intergenic regions, are shown as 5% windows from 5′ to 3′.

The surprising stability of RNA in the hair shaft led us to examine and identify how RNA read coverages differ between viable and non-viable tissue. We thus compared RNA libraries of the hair shaft to viable NHEK using the same sequencing platform. First, we found that lineage-restricted hair keratin genes were highly enriched in the hair shaft library relative to NHEK [34], [35] (Fig. 4A). Many of the neighboring non-hair shaft keratin genes were absent from the hair shaft library. Comparing the expression of six different cell types in the hair follicle, we concluded that RNA of the human hair shaft library predominantly originate from transcripts of the three hair committed cell types (Fig. 4B, C). We also considered whether RNA from a non-viable source might demonstrate different patterns of intragenic region coverage compared to viable keratinocytes (Fig. 4D). Here, we found a similar pattern of coverage of intragenic regions, e.g. 5′ untranslated regions (UTR), exonic, intronic, and 3′ UTR (Fig. 4E). In both RNA libraries, intronic transcripts, which include 5S rRNA, were the most abundant. Exonic sequences of both libraries revealed similar patterns of coverage, with the exception that the 5′ regions of viable keratinocytes (32.5%) were relatively more enriched than in the hair shaft RNA library (24.2%). Thus, the abundance of detected transcripts and the high degree of similarity in the persistent RNA of the non-viable hair shaft and viable keratinocytes indicate that RNAs are inefficiently targeted by apoptosis during hair.

### miRNAs and mRNAs Are Stable In Distal Hair Segments

**Figure 5:**
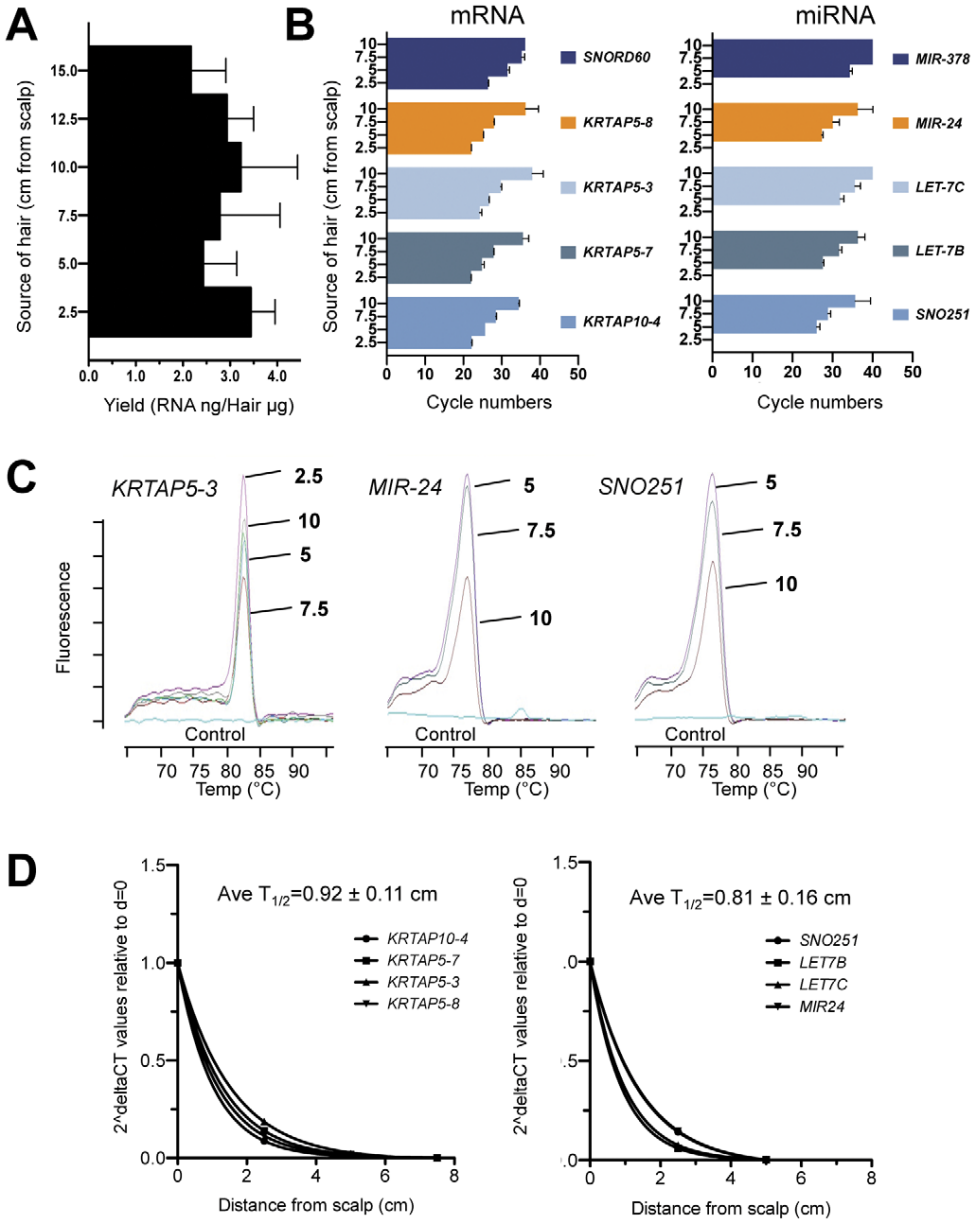
Stability of mRNA and miRNA in hair shaft at varying distances from scalp. (A) Total RNA yields from hair shaft at varying distances from the scalp as detected by A260 absorbance. (B) Detection of specific mRNAs and miRNAs identified from hair shaft library at varying distances from scalp, indicated in cycle numbers. Higher cycle numbers reflect reduced levels of detection, particularly at 10 cm. All experiments shown demonstrate at least three replicates and at least two individuals. (C) Melting peaks of *KRTAP5-3*, *MIR-24* and *SNO251* as an example of amplified mRNA, miRNA, and snoRNA products from 2.5 to 10-cm segments, respectively. (D) Calculated average half-lifes of mRNAs vs. miRNAs based on cycle numbers using non-linear fit one-phase decay modeling. Because growth rates of hair were not directly assessed in these studies, the distance of hair sampled from scalp was used as a marker of relative time.

During hair growth, cells are continuously incorporated into the hair shaft such that as the hair elongates, the progressively older segments of hair are pushed further distally from the scalp. Given the remarkable stability of RNA following cornification in the hair shaft inferred from the large number of detected transcripts and the many similarities to viable keratinocytes, we investigated the stability of RNA at varying distances, using the the scalp as the point of origin. Strands of hair from two individuals were collected, aligned and divided into 2.5 cm segments. We found that RNA could be consistently isolated even at distant regions of the hair shaft (Fig. 5A). At increasing distances, higher cycle numbers were required to detect miRNA and mRNA target genes by real-time PCR, indicating decreased abundance of specific RNA transcripts (Fig. 5B). Nevertheless, even at these higher amplification cycles, specific products were obtained and could be confirmed by the melting curve (Fig. 5C) and gel electrophoresis (not shown). Decay of mRNA and miRNA detection was estimated by examining the increasing cycle numbers over a relative measure of time, in this case, hair length (Fig. 5D). We find that a two-fold reduction of mRNAs and miRNAs occurs over the length of 0.92±0.11 cm and 0.81±0.16 cm, respectively. These studies suggest that the conditions that allow RNAs to be stable during hair formation persist even months after cornification and provide insights into the natural decay of RNAs in the hair.

## Discussion

In this study, we demonstrate that as a direct remnant of previously living cells, hair retains a vast amount of transcriptional data reflective of incomplete degradation of RNA following apoptosis. In addition to hundreds of miRNAs and snoRNAs, sequencing of hair RNA identified 7,193 unique mRNAs, or an equivalent to over a quarter of all genes in the human genome. Hair RNAs potentially reflect months of expression data temporally deposited along the length of the hair shaft and may be useful for investigations of gene expression and biomarker discovery.

As a model for studying differentiation and cell death, the hair provides insights into the extent to which RNAs are targeted for degradation during cornification. The numerous RNAs identified in the hair shaft suggest that RNA degradation is highly inefficient during cornification. In support of this hypothesis, read coverage between hair shaft versus viable keratinocyte RNA libraries exhibited remarkable similarities in coverage of intragenic regions with small biases toward reduced exonic coverage in hair shaft. Several RNases were detected in the hair shaft RNA library, including *RNASE4, 7, 12, 13, RNASEN*, and *RNASET2*, indicating that lack of RNases do not account for the persistence of RNAs in hair. RNA stability in hair might involve other mechanisms such as sequestration of RNases, presence of endogenous RNase inhibitors, protection of RNA by ribonucleoprotein particles or low water content in hair [36]. Understanding the contribution of these potential mechanisms may be important in improving RNA detection and stability in future hair RNA expression studies. The removal of DNA versus RNA during apoptosis may play distinct roles in different tissues [3]. In erythrocytes, corneocytes, and lens fiber cells, where nuclei and DNA are removed during terminal differentiation, persistence of RNA may be important in maintaining cell function. Whether incompletely removed RNA in hair shaft serves a functional purpose is unknown. It is unclear if mRNAs identified in the hair shaft are available for translation as the degree of fragmentation and survival of translational machinery were not examined in this study. In addition, studies aimed at determining whether RNA persists in other cornified tissues such as the nail could be of great importance for studying of diseases that affect localized body sites, such as cancer, infection, injury and exposures.

The discovery of stable mRNAs and miRNAs distant in the hair shaft from the scalp overcomes a formidable obstacle in the application of RNA diagnostics. Currently, RNA studies based on tissue biopsy and phlebotomy are vulnerable to RNA degradation [37]. In the current study, we found that older, more distal regions of hair still contain detectable mRNAs and miRNAs. These findings have several implications. First, the stability of RNA in older portions of hair suggests that RNA may be stable in hair over many months. This attribute differs greatly from other tissues and blood and potentially resolves a problem in storage and transportation of RNA, necessary for RNA-based diagnostic or biomarker studies. Second, temporal changes in gene expression either physiologic or induced by chemical, drug or disease might be stored co-linearly along the length of the hair shaft. Use of this spatiotemporal pattern of RNA deposition might provide a novel approach to studying the natural course or inciting events of disease. In addition to these characteristics, the continuous replacement of hair and its ease of access are advantages to developing diagnostic approaches based on hair RNA.

While possibly ideal for many types of biomedical studies, the use of hair RNA for molecular studies and diagnosis currently has several limitations. At this time, the amount of variation in RNA expression between different individuals, ages, and genetic backgrounds are not known. In addition, differential growth rates of hair in individuals due to differences in genetic background, age, and other factors obscure accurate measurements of time. Recent studies indicate that growth of human hair may vary from 1.3 to 2.2 months per centimeter in different individuals [3]. More accurate measurements might be made possible with the identification of cyclically expressed genes, which could used to normalize differential growth rates [38]. An additional limitation is that since new cells are added to the hair shaft only during active hair growth, it is not yet known what the effects different stages of the hair cycle might have on the pattern and stability of RNA in the hair. In this case, identification of transcripts representative of the final stages of the hair cycle might be required to determine whether retained transcripts reflect specific portions of the hair cycle. Lastly, because the characteristics that contribute to RNA stability in the hair are unknown, it is possible that RNA stability varies in individuals. These unknown aspects may bias the results of RNA detection in the hair.

The use of parallel sequencing of small RNAs provides an atlas of residual transcripts in humans of different genders, populations, and hair type. Sequencing technology provides several informative features valuable to molecular diagnostic studies including transcriptional orientation, multiple means of validation of expression and quantity of transcripts, and sequence data [39], [40]. Sequence data also provide a significant source for the identification of genetic polymorphisms, detection of allele-specific expression differences, and somatic mutations. The extent to which variant alleles are present in hair shaft RNA was not explored in this study. Current obstacles to use of this data for allele-specific studies include the possibility of chemical changes in nucleotides, e.g. cytosine deamination [41], and incorporation errors inherent to RNA polymerases [42]. Further studies are needed to assess the impact of these factors on the fidelity of hair RNA sequences.

In the past, hair analysis has aided in the diagnosis of inherited and acquired diseases [14], [43], [44] and as a phenotypic marker of medication and chemotherapeutic response [45]. Thus while the discovery of post-apoptotic RNA stability in hair provides insights into programmed cell death, these findings also greatly expand the possible applications of hair in medical discoveries in the clinic and the lab. The unique properties of the linear growth of the hair serve as a record of gene activity during organ growth and of the individual's own history. This record may be of great value for studies in organ development, evolution, genetic variation, and population genomics.

## Materials and Methods

### Animal and Human Specimens

Mouse lines originated from a CF-1 mixed genetic background (Charles River). Human samples were unidentified and pooled. The demographic profiles are shown in Supplemental Methods. Normal human epidermal keratinocytes were grown in EpiLife culture media (Cascade Biologics, Portland, OR) containing 0.06 mM calcium and defined growth supplement. All experiments and informed consents were performed and approved according to the institutional guidelines established by the University of California, San Diego, Institutional Animal Care and Use Committee and the University of California, San Diego, Human Research Protection programs, Protocol ID #091646. Written informed consents were obtained from each participant of the study.

### Hair collection, storage, and RNA extraction

For mouse studies, hair from 3-week old animals was trimmed and stored in RNAlater (QIAGEN) solution at −80°C. Hair was visually inspected to verify absence of hair bulbs, which contain viable cells. For human studies, hair was trimmed at a distance of 5 mm from the scalp. Hair was washed in 70% ethanol and water. Two methods were used for RNA extraction. During initial studies in mouse hair and human hair shaft RNA-sequencing, Trizol reagent (Invitrogen) was utilized in combination with mechanical disruption with 1.0 mm zirconia beads (BioSpec Products, Inc.). Subsequently, improved extraction was obtained with the addition of 0.1 M dithiothreitol (DTT) to Trizol reagent or a urea-based RNA extraction buffer [46]. The improved yield of RNA presumably results from the effects of a reducing agent on the highly disulphide crosslinked hair tissue. A more detailed protocol is described in Supplemental methods.

### Real-time PCR analysis for mRNAs, snoRNAs, and microRNAs

Primer sequences and miRNA probes are detailed in Supplemental Methods. Primers for mRNAs were designed using Primer3 [47]. Reverse transcription and real time PCR were performed with Maxima First Strand cDNA Synthesis Kit (Fermentas) and Maxima SYBR Green (Fermentas), respectively. For microRNA assays, cDNA was prepared using a RT^2^ miRNA First Strand Kit (QIAGEN) and amplified with stem-loop primers from SABiosciences/QIAGEN (Table S4) using RT^2^ SYBR® Green qPCR mastermix (QIAGEN). Real-time PCRs were performed on an ABI 7300 Real Time PCR system and a Roche Lightcycler 480. A comparative C_T_ method was used for quantitative analysis using *snoRNA-251* in mouse microRNAs samples, *Gapdh* for mouse mRNAs. Half-life of mRNAs and miRNAs determined using 2∧deltaCT values normalized to levels at site of origin and Prism (Prism 5, Version 5.0d) non-linear fit first-order decay modeling.

### RNA Sequencing and Analysis

Fifty hair shafts, equivalent to 30 mg, were utilized to prepare total RNA using the above methods. One microgram of total RNA isolated from hair was used to generate a small RNA library. Adapter ligation, reverse transcription, amplification, gel extraction and sequencing were performed, according to Illumina protocols for miRNA-Seq (v1.5.0) (Illumina). Sequencing was carried out on an Illumina Genome Analyzer IIx. Genome Analyzer Pipeline was used to generate a FASTQ file from raw RNA-seq data, and quality scores were offset by 64 following Solexa-1.3+ standards. FASTX-Toolkit package, hg18 genome alignment with BOWTIE, was used for pre-processing and alignment. Parameters for alignment are detailed in Supplemental methods, and a list of human hair shaft transcripts are provided in Table S5. Computational processes were carried on Triton Resource at the San Diego Supercomputer Center at UCSD, using a single node of 32 processors and 512GB of RAM. For NHEK to hair shaft RNA library comparisons, representative coverages of intragenic regions in REFSEQ were compared. Each gene in REFSEQ was segmented into annotated 5′-UTR, introns, exons, and 3′-UTR regions; these regions were further broken up into windows of 5% of the length of the region. The hair shaft library was aligned to these regions, and the total coverage of all 5% windows for each region type in each gene was plotted, generating a summary of overall coverage patterns in intragenic regions. Functional and compound associations were performed using Set-Distiller batch tool [33] and Mouse Genome Informatics [48]. Further methods are described in Methods S1 and accompanying References S1.

## Supporting Information

Table S1 Annotation and reads counts of mRNA, miRNA, snoRNAs (>200 reads).
(PDF)

Table S2 Representation of Genetic Association Database transcripts in hair.
(PDF)

Table S3 Representation of Novoseek compound-genetic pathways in hair.
(PDF)

Table S4 Primers and reagents used in PCR analysis.
(PDF)

Table S5 Annotated reads with >10 reads.
(XLS)

Methods S1 Supplemental methods.
(DOC)

References S1 Supplemental references.
(DOC)
