# Supplementary material for: The Post-Apoptotic Fate of RNAs Identified Through High-Throughput Sequencing of Human Hair
Source: PLoS One. 2011 Nov 16;6(11):e27603. doi: 10.1371/journal.pone.0027603 (PMC3218001; doi:10.1371/journal.pone.0027603)
Supplement: Table S1 — Annotation and reads counts of mRNA, miRNA, snoRNAs (>200 reads). (PDF) [file pone.0027603.s001.pdf]

Table S1
Lefkowitz et al.

Human hair shaft mRNAs

mRNA name	Read Count	mRNA name	Read Count	mRNA name	Read Count
CYP46A1	28,457	*C1orf213*	2,514	*AHNAK2*	617
SHSA2	28,009	*KRTAP12-1*	2,469	*TPM3*	615
KRTAP5-4	21,063	*ABCA13*	2,202	*C17orf91*	596
KRTAP5-8	18,948	*NEAT1*	2,142	*DNMT3B*	586
KRTAP5-10	18,718	*KRTAP12-2*	2,022	*PABPC3*	582
KRTAP5-7	17,881	*TRPV2*	1,944	*CNO*	579
KRTAP5-5	17,709	*KRTAP19-1*	1,871	*EXPH5*	549
KRTAP5-3	15,045	*SSH1*	1,522	*KRTAP4-12*	533
KRTAP5-2	14,959	*RMRP*	1,428	*PNOC*	524
KRTAP5-1	13,637	*SLCO1C1*	1,342	*KRTAP24-1*	508
KRTAP10-11	13,007	*ASB11*	1,241	*C1orf204*	505
KRTAP10-4	11,224	*FLG2*	1,219	*KRTAP4-8*	495
MALAT1	10,832	*EN1*	1,179	*MYST4*	489
KRTAP10-9	10,635	*MLL2*	1,153	*FAM22G*	482
KRTAP5-11	10,398	*SCARNA10*	1,072	*KRTAP4-11*	458
KRTAP10-2	10,129	*ATCAY*	1,048	*KRTAP4-9*	456
KRTAP10-6	9,075	*C15orf52*	985	*DHX36*	433
KRTAP10-5	8,352	*KRTAP9-8*	961	*FAM43A*	433
C17orf44	8,224	*LRRC15*	959	*TUBB2C*	427
KRTAP10-1	7,971	*KRTAP19-3*	937	*DSP*	425
KRTAP10-7	7,863	*UBC*	923	*KRT15*	424
C17orf68	7,595	*LOC115110*	921	*SCARNA11*	419
KRTAP10-12	6,925	*KRTAP9-3*	911	*KRTAP3-3*	416
KRTAP5-6	6,163	*KRTAP9-4*	889	*WNT2B*	407
KRTAP10-3	6,041	*AHNAK*	831	*ZNF263*	389
KRTAP5-9	5,552	*CHAC1*	792	*SCRIB*	382
ARHGAP20	5,344	*RPL32*	781	*KRT5*	378
KRTAP17-1	5,151	*SNX3*	771	*KRTAP4-7*	374
FLG	4,348	*IRF2BP1*	719	*LOC80154*	373
KRTAP10-10	3,848	*RBM33*	692	*ODZ1*	372
VSIG8	3,591	*TMC5*	682	*GOLGA2P2*	371
KRTAP10-8	3,433	*IRGQ*	666	*GOLGA2P3*	371
CDK6	2,969	*KRTAP9-9*	648	*KRT6C*	363
TBC1D4	2,815	*KRT31*	632	*CCDC151*	361
KRTAP10-7	2,561	*KRT33B*	631	*KRT33A*	361

Table S1
Lefkowitz et al.

Human hair shaft mRNAs

mRNA name	Read Count
KRT6B	357
KRT86	356
LOC642006	351
KRTAP4-4	348
KRT6A	343
KRTAP4-3	333
VTRNA1-1	332
KRT14	331
JAG2	327
SORT1	325
COL7A1	323
KRT16	323
SCD	318
PPTC7	313
DAPK2	309
KRTAP1-5	309
YAF2	305
LOC388152	302
PPP1R15A	301
FOXD3	298
USMG5	297
LATS2	296
MMRN2	293
COL20A1	285
PIGS	285
VTRNA1-3	285
KRT34	284
BOK	282
SLC15A2	282
SOS1	278
KRTAP19-5	277
PAR5	277
KRT16P2	276
S100A3	274
GUCY1A2	269

mRNA name	Read Count
LAMA5	268
RDH8	263
SPATA13	263
KRT32	259
PSORS1C2	256
SFXN3	256
PABPC1P2	255
HTATIP2	254
KRTAP3-2	249
VSTM2A	248
ZNF473	248
KRT81	245
KRTAP1-1	243
KRT83	239
KRTAP4-2	238
DIP2C	237
FAM36A	235
KLF13	228
KRT36	227
C12orf66	225
DGCR9	225
KRTAP4-5	225
KRT85	224
ZNF70	222
LEMD2	215
BHLHE41	214
MRPS5	213
SBSN	213
LAMB2	212
LOC150622	211
PABPC1	208
PCDHGA6	207
KRTAP26-1	205
BEAN	204
HSP90AA1	204

mRNA name	Read Count
SCARNA3	203
SLC6A18	203
RFK	202
CLASP1	201
PIP5K1B	201
SURF1	201

Table S1
Lefkowitz et al.

Human hair shaft microRNAs

miRNA name	Read Count	miRNA name	Read Count	miRNA name	Read Count
MIRLET7A1	789,964	*MIR148A*	11,493	*MIR143*	*1,643*
MIRLET7A3	783,705	*MIR320A*	11,477	*MIR944*	1,615
MIRLET7A2	777,499	*MIR320B2*	9,901	*MIR17*	1,584
MIRLET7B	672,415	*MIR320B1*	9,625	*MIR193A*	1,492
MIRLET7F2	547,114	*MIR26B*	9,498	*MIR1246*	1,407
MIRLET7E	511,541	*MIR125A*	7,099	*MIR106A*	1,405
MIR203	294,637	*MIR99B*	6,726	*MIR29A*	1,335
MIRLET7G	270,697	*MIR181B2*	6,594	*MIR425*	1,334
MIR24-1	113,886	*MIR181B1*	6,358	*MIR186*	1,249
MIR24-2	113,793	*MIR320C2*	5,326	*MIR221*	1,093
MIR200C	104,858	*MIR141*	5,192	*MIR320D2*	1,015
MIRLET7D	83,447	*MIR196A2*	5,146	*MIR195*	1,005
MIR200B	31,859	*MIR196A1*	5,052	*MIR106B*	942
MIR1826	28,393	*MIR196B*	4,993	*MIR486*	909
MIR146B	26,319	*MIR183*	4,943	*MIR130A*	898
MIR205	21,574	*MIR125B2*	4,468	*MIR429*	892
MIR30A	18,398	*MIR1974*	4,404	*MIR149*	793
MIR17HG	18,052	*MIR1974*	4,404	*MIR1-1*	787
MIR146A	17,662	*MIR191*	4,369	*MIR1-2*	786
MIR23A	17,391	*MIR125B1*	4,159	*MIR15B*	771
MIR103-2	16,188	*MIR92B*	3,733	*MIR215*	761
MIR103-1	16,173	*MIR200A*	3,564	*MIR451*	757
MIR181A2	15,574	*MIR99A*	3,383	*MIR130B*	756
MIR30E	15,135	*MIR100*	3,296	*MIR224*	652
MIR181A1	14,954	*MIR140*	3,131	*MIR22*	592
MIR30D	14,343	*MIR16-2*	2,965	*MIR30B*	577
MIR92A1	13,834	*MIR16-1*	2,961	*MIR886*	558
MIR182	13,098	*MIR744*	2,961	*MIR193B*	541
MIRLET7I	13,079	*MIR320C1*	2,751	*MIR598*	535
MIR142	12,494	*MIR181D*	2,515	*MIR375*	533
MIR21	12,486	*MIR30C2*	2,369	*MIR222*	532
MIR27B	12,408	*MIR30C1*	2,353	*MIR629*	532
MIR27A	11,918	*MIR101-2*	2,204	*MIR455*	484
MIR26A1	11,586	*MIR101-1*	2,198	*MIR664*	483
MIR26A2	11,559	*MIR20A*	1,978	*MIR144*	415

Table S1
Lefkowitz et al.

Human hair shaft microRNAs

miRNA name	Read Count
MIR155	406
MIR155HG	406
MIR29B2	401
MIR29B1	397
MIR10A	355
MIR10B	346
MIR210	337
MIR374A	318
MIR339	316
MIR15A	305
MIR769	302
MIR1307	297
MIR342	281
MIR19B2	278
MIR19A	275
MIR197	264
MIR508	247
MIR128-2	239
MIR128-1	238
MIR206	238
MIR1977	229
MIR145	215
MIR96	211
MIR194-2	209
MIR194-1	202

Human hair shaft snoRNAs

snoRNA name	Read Count
SNORD60	714,033
SNORD104	26,901
SNORD43	14,037
SNORD69	12,526
SNORD100	9,201
SNORD20	8,052
SNORD62A	7,804
SNORD62B	7,804
SNORD62A	7,793
SNORD62B	7,793
SNORA61	5,157
SNORD66	3,781
SNORD78	3,744
SNORD93	3,069
SNORD31	2,992
SNORD89	2,729
SNORD44	2,395
SNORD63	2,304
SNORD95	1,874
SNORD94	1,859
SNORD27	1,535
SNORD48	1,528
SNORD48	1,527
SNORD68	1,406
SNORD90	1,379
SNORD21	1,198
SNORD26	976
SNORD102	893
SNORD79	844
SNORA7A	779
SNORD2	774
SNORA7B	769
SNORD119	768

snoRNA name	Read Count
SNORD57	754
SNORD33	747
SNORD81	644
SNORD28	642
SNORD30	624
SNORD74	572
SNORD15A	555
SNORD71	506
SNORA36B	483
SNORD46	463
SNORD82	446
SNORD67	433
SNORD6	432
SNORA70	352
SNORA70B	337
SNORA62	331
SNORD97	315
SNORD83A	279
SNORD64	277
SNORD42A	276
SNORD48	252
SNORD55	247
SNORA45	246
SNORD1B	241
SNORD7	216
SNORD96B	214
SNORD38B	206
