# Supplementary material for: The Post-Apoptotic Fate of RNAs Identified Through High-Throughput Sequencing of Human Hair
Source: PLoS One. 2011 Nov 16;6(11):e27603. doi: 10.1371/journal.pone.0027603 (PMC3218001; doi:10.1371/journal.pone.0027603)
Supplement: Table S2 — Representation of Genetic Association Database transcripts in hair. (PDF) [file pone.0027603.s002.pdf]

Table S2
Lefkowitz et al.

Gene Symbol	Disease Association	OMIM	PMID
ABCA13	Schizophrenia, Bipolar, Depression susceptibility	607807	
ATCAY	Cayman Ataxia	608179	
BEAN	Spinocerebellar ataxia 31	612051	
BHLHE41	Short Sleep Syndrome	606200	
CNO	Hermansky-Pudlak Syndrome	203300	
COL7A1	Dystrophic epidermolysis bullosa (Hallopeau–Siemens)	226600	
CYP46A1	Alzheimer's disease risk		17192785
DNMT3B	Immunodeficiency-centromeric instability-facial anomalies	242860	
DSP	Dilated cardiomyopathy with woolly hair and keratoderma	605676	
	Arrhythmogenic right ventricular dysplasia/cardiomyopathy	607450	
FLG	Ichthyosis vulgaris, atopic dermatitis	146700, 603165	
KRT14	Epidermolysis Bullosa Simplex	131760, 601001	
KRT16	Pachonychia congenita	167210	
KRT5	Epidermolysos Bullosa Simplex	131760, 601001	
KRT6A	Pachonychia congenita	167210	
KRT6B	Pachonychia congenita	167210	
KRT81	Monilethrix	158000	
KRT83	Monilethrix	158000	
KRT86	Monilethrix	158000	
LAMB2	Congenital nephrotic syndrome (Pierson Syndrome)	609049	
LEMD2	Carbamazepine adverse reactions		16538176
MLL2	Kabuki Syndrome	147920	
PIP5K1B	Chronic renal disease susceptibility		20383146
PSORS1C2	Psoriasis susceptibility	177900	
RDH8	Macular Degeneration, Age-Related 2	153800	
RMRP	Catilage-Hair hypoplasia	250250	
SORT1	Coronary artery disease	613589	21378990
	Dyslipidemia	613589	
SOS1	Noonan syndrome	163950	
SURF1	Leigh syndrome (Infantile subacute necrotizing encephalopathy)	256000	
TPM3	Nemaline myopathy	609284	
