# Supplementary material for: The Post-Apoptotic Fate of RNAs Identified Through High-Throughput Sequencing of Human Hair
Source: PLoS One. 2011 Nov 16;6(11):e27603. doi: 10.1371/journal.pone.0027603 (PMC3218001; doi:10.1371/journal.pone.0027603)
Supplement: Table S3 — Representation of Novoseek compound-genetic pathways in hair. (PDF) [file pone.0027603.s003.pdf]

Table S3
Lefkowitz et al.

Gene Symbol	Novoseek Compounds
AHNAK	Calcium
BHLHE41	VEGF
BOK	Progesterone, Estradiol, Thymidine, Oxygen, Estrogen, Retinoic Acid, VEGF
CDK6	Indole-3-Carbinol, 3,3'-Diindolylmethane, Silicon Phthalocyanine, EB 1089, Fisetin, Silymarin, Roscovitine, 17-(Allylamino)-17-Demethoxygeldanamycin, Indole, Lovastatin, Thymidine, EGCG, Butyrate, Threonine, Rottlerin, Flavonol, Arsenite, Berberine, Monensin, Flavone, Gefitinib, Tyrosine, Apigenin, Trastuzumab, Resveratrol, Herbimycin A, 1,25 Dihydroxy Vitamin D3, Phorbol 12,13-Dibutyrate, TGF Beta1, Retinoic Acid, LY294002, Estrogen, Calcium, Oxygen, Proline, Ionomycin, Alanine, Progestin, Cisplatin, Tamoxifen, Serine, Dihydrotestosterone, Valine, Dexamethasone, Rapamycin
COL20A1	Hydroxylysine, Hydroxyproline, Gold, Tyrosine
COL7A1	Arginine, Valine, Serine, Cysteine, Chloramphenicol
CYP46A1	24S-hydroxy-cholesterol, Lathosterol, Cholesterol
DAPK2	Threonine, Serine
DNMT3B	5-aza-2'deoxycytidine, 5-Methylcytosine, Cytosine, Zebularine, Arsenite, Folate, Tamoxifen, Homocysteine, Oligonucleotide, Zinc, Estrogen
DSP	Asulam, N,N-Dimethylformamide, TGF Beta1, Ryanodine, Retinoic Acid, Calcium, Sucrose, Steroid, Progesterone, Tyrosine, 12-O-Tetradecanoylphorbol 13-Acetate, Serine, Acetone, Thapsigargin, DMSO
FLG	Citrulline, Retinoic Acid, Calcipotriol, Dithranol, Retinoid, Mecamylamine, Serine, Ceramide, Arginine, Calcium, GF 109203x, Allergens, Histidine, Lipid, Arsenate, Bromodeoxyuridine, Hematoxylin, Nitric Oxide, Nickel, Fibrinogen, Steroid, Latex, Tyrosine, Urea, Nicotine, Cysteine, Calcitriol, Infliximab, Salicylic Acid, Ascorbic Acid, SU5402, SU6668, Tyrosine, Heparan Sulfate, Heparin, VEGF, GNRH Phosphotyrosine, Apicidin, Sodium Chlorate, Imatinib, SU5416, Pd 98,059, Saporin, Suramin, Chlorate, Thymidine, SB 203580, Suberoylanilide Hydroxamic Acid, Dextran Sulfate, Histamine, Phenylalanine, Agar, Steroid, Calcium, LY294002, Glutamate, Lactate, Hydrogen, Lipid, Nitric Oxide, Glucose, Ribonucleic Acid, Lysine, Oligonucleotide, Nacl, Pyruvate, Atp, Forskolin, Fibrinogen, Chondroitin Sulfate, Progesterone, Threonine, Zinc, Glyceraldehyde 3-Phosphate, Phosphatidylinositol, Bromodeoxyuridine, 12-O-Tetradecanoylphorbol 13-Acetate, Methionine, Leucine, Estrogen, Adenylate, Genistein, Testosterone, Polysaccharide, Serine, Retinoic Acid, Cysteine, Dopamine, Paclitaxel, H2O2
HSP90AA1	Geldanamycin, 17-(Allylamino)-17-Demethoxygeldanamycin, Radicicol, 17-Amino-17-Demethoxygeldanamycin, 17-DMAG, LBH-589, Novobiocin, Molybdate, Steroid, Herbimycin A, Suberoylanilide Hydroxamic Acid, Atp, 5-Methoxy-1,2-Dimethyl-3-(4-Nitrophenoxymethyl)Indole-4,7-Dione, Dimethyl Pimelimidate, Rifabutin, Sodium Molybdate, Tyrosine, Amp-Pnp, Coumermycin, Lactacystin, Threonine, Progesterone, MG 132, Trastuzumab, Rapamycin, Depsipeptide, Tacrolimus, Sodium Arsenite, Quinone, Estrogen, Adp, Tcdd, Bortezomib, Imatinib, Serine, Arsenite, Phosphatidylinositol, VEGF Hydroxamate, Sulforhodamine B, 4-Hydroxytamoxifen, Methylmethanethiosulfonate, Nitric Oxide, Chlorothalonil, Nitrit, Pyrazole, LY294002, ATPgammas, Okadaic Acid,

Table S3
Lefkowitz et al.

Gene Symbol	Novoseek Compounds
HSP90AA1 (cont'd)	Hydroquinone, Cisplatin, Curcumin, Nonidet-P40, N-Acetylcysteine, Testosterone, Oxime, Coumarin, Mifepristone, Calpeptin, Leptomycin B, Cycloheximide, Quercetin, Citrate, Glyceraldehyde 3-Phosphate, Purine, Tungstate, Nocodazole, Polyacrylamide, Dexamethasone, Cysteine, Doxorubicin, Arachidonic Acid, Glutamate, Tamoxifen, Adenylate, Paclitaxel, Lactate, Glutamine, Morphine, Colcemid, Heparin, Alanine, Amphotericin B, Cytarabine, Oxaliplatin, Arginine, PGE2, N-Ethylmaleimide, Atorvastatin, Prostacyclin, Genistein, Anti-Fungal, Spironolactone, Butyrate, Corticosterone, 4-Hydroxynonenal, Neomycin, 5-Fluorouracil, Retinoic Acid, Glycerol, Etoposide, Dihydrotestosterone, Adenine, Carbamazepine, Camptothecin, Docetaxel, Sb 203580, Erlotinib, Thapsigargin, H2O2, Deae, Cyclosporin A, Vitamin D
KLF13	RANTES, Zinc, Proline, Alanine
LAMA5	Heparan Sulfate, Carbohydrates, Heparin, VEGF
LAMB2	Tripeptide, Acetylcholine, Heparin, Calcium
LATS2	Threonine, Serine
MYST4	Zinc
PABPC1	pABC, Adenylate
PIP5K1B	Phosphoinositide, Lipid
PNOC	Nalbzoh, Naloxone, Tertiapin, Etorphine, Damgo, Naltrindole, [3h]Diprenorphine, Carbetapentane, Norbinaltorphimine, Octadecaneuropeptide, Morphine, Opiate, Npff, Dpdpe, Naltrexone, Rimcazole, Buprenorphine, Tetrapeptide, Anandamide, Aconitine, Capsaicin, Alpha-Aminoisobutyric Acid, Amphetamine, Piperidine, Trifluoroacetic Acid, Baclofen, Guanethidine, Cyclic Amp, Cocaine, Sufentanil, Gaba, Norepinephrine, Forskolin, Atropine, Hexamethonium, 5-Hydroxytryptamine, Dopamine, Nmda, Glutamate, Adenylate, Potassium, Lipid, Indole, Gtp, Sodium, Prostaglandin, Nitric Oxide, Steroid, Calcium, Progesterone, Tyrosine, Amide, Superoxide, Alanine, Estrogen, Pge2, Histamine, Acetylcholine
PPP1R15A	O6-Benzylguanine, Thapsigargin, Okadaic acid, Methylmethanesulfonate, Tunicamycin, Peroxynitrite, Cisplatin, Threonine, Carboplatin, Lactate, Methionine, Serine, VEGF
RAP1A	GTP, GDP, Guanosine, Forskolin, Cyclic Amp, Geranylgeranyl Pyrophosphate, Tyrosine, Mevalonate, Perillyl Alcohol, Guanine, LY294002, Calcium, Asparagine, Risedronate, Phorbol, Rolipram, Phosphatidylinositol, Farnesyl Diphosphate, Adenylate, Nadph, GF 109203X, Prostacyclin, IBMX, Threonine, FMLP, Serine, Nocodazole, Phosphoinositide, Ionomycin, Isoproterenol, CGMP, Lipid, PGE2, 12-O-Tetradecanoylphorbol 13-Acetate, Oxygen, Agar, Nitric Oxide, Superoxide, Carbachol, Arginine, PGE1, VEGF, Glutamine, NMDA
RDH8	Vitamin A
RFK	Riboflavin, Flavin Mononucleotide, Flavin-Adenine Dinucleotide, Flavin, MGADP, ATP
RPL32	Glyceraldehyde 3-phosphate
S100A3	Calcium, Zinc
SCD	Stearoyl-Coa, Sterculic Acid, Fatty Acid, Palmitoleate, Linoleic Acid, Oleic Acid, Stearic Acid, Palmitoleic Acid, Sterol, Acetyl-Coa, Lipid, Palmitate, 3-Hydroxy-3-Methylglutaryl-Coa, Alpha-Linolenic Acid, Triacylglycerol, Docosahexaenoic Acid, Acyl-Coa, Cholesterol, Arachidonic Acid, Glycerol 3-Phosphate, Ceramides, Phospholipid, Oxo, Eicosapentaenoic Acid, Methane, Rosiglitazone, Troglitazone, Glucose, Cholesterol Ester, Farnesyl Diphosphate, IBMX, LY294002, Dexamethasone, ATP, Vitamin-E, Oxygen, P003, Iron, Phosphatidylinositol, Histidine, Carbohydrates, Phosphatidylcholine, Retinoic Acid, Carbon, Alpha Tocopherol, Vitamin A, Clozapine, Haloperidol

Gene Symbol	Novoseek Compounds
SLC15A2	Glycylsarcosine, Tripeptide S, Valacyclovir, 5-Aminolevulinic Acid, Tripeptide, Ganciclovir, Nucleoside, Histidine
SORT1	Tyrosine, Glutamate
SOS1	Tyrosine, Menadione, Phosphatidylinositol, GTP, Serine, Lipid
SURF1	Lactate, Pyruvate, Bicarbonate, Oxygen
TBC1D4	Phosphatidylinositol, Glucose
TPM3	Tyrosine, Calcium
TRPV2	Capsaicin, Calcium
UBC	Finrozole, Porphobilinogen, Dexamethasone
