# Supplementary material for: The Post-Apoptotic Fate of RNAs Identified Through High-Throughput Sequencing of Human Hair
Source: PLoS One. 2011 Nov 16;6(11):e27603. doi: 10.1371/journal.pone.0027603 (PMC3218001; doi:10.1371/journal.pone.0027603)
Supplement: Table S4 — Primers and reagents used in PCR analysis. (PDF) [file pone.0027603.s004.pdf]

Table S4. Primers and reagents used in real-time PCR analysis

MicroRNA	Catalog Number	Source
mmu-mir-199	MPH01234A-200	Sabiosciences/Qiagen
mmu-mir-203	MPM01435A-200	Sabiosciences/Qiagen
mmu-mir-212	MPM00590A-200	Sabiosciences/Qiagen
snoRNA-251	MPM01664A-200	Sabiosciences/Qiagen
hsa-MIR-LET7B	MPH00002A-200	Sabiosciences/Qiagen
hsa-MIR-LET7C	MPH00003A-200	Sabiosciences/Qiagen
hsa-MIR-24	MPH01234A-200	Sabiosciences/Qiagen
hsa-MIR-378	MPH01284A-200	Sabiosciences/Qiagen

Gene		Forward		Reverse
Krt34	Ex6/7	GGAGAGCGAGGACTGCAACCTC	Ex7	ACGCTTTGAGCTGCCGCAAGG
Krt32	Ex6	GGCCTGCTGGAGAGTGAGGACAG	Ex7	CACAGACGGTGCGGGATACCCC
Krt35	Ex1	TGCTGTGGCCATGGCTTCCAAA	Ex1	GAGTGGATCCCCCTCCGGCTT
Bmp6	Ex6	CTGCGCACCAACCAAACTGAA	Ex6	TTGGGGGAGGCGAACATTAGGTA
Sh3d19	Ex1	GATGACGTATTGCCCACCTC	Ex2	GTTTGGCTGGGATTCGTATT
Sox2	Ex1	TACTGGCAAGACCGTTTTCGTG	Ex1	CTCGGCAGCCTGATTCCAATA
SNORD60		GCACGTGCAGTTTTCATACG		TGTGATGAATTGCTTTGACTTCT
KRTAP5-8	Ex1	AAGGGCCCAGGCTCAGGGAG	Ex1	GGAGCAGGTGAGAGGGAGGTGT
KRTAP5-3	Ex1	AGCCACAAGAACCGCAGCCC	Ex1	GCTGCTCCTCCAGCTGTGGC
KRTAP5-3	Ex1	TGAGGAGCAGCAGCAGGGCT	Ex1	GGGCTGCGGTTCTTGTGGCT
KRTAP5-7	Ex1	GGCTCTGGACTCAGGTCTCA	Ex1	GGACCCTGAGCAGTGGTTT
KRTAP10-4	Ex1	GCAAGACTGTCTGCTGCAAG	Ex1	AGCATGAAGAATCCCCACAG
