# Supplementary material for: The Post-Apoptotic Fate of RNAs Identified Through High-Throughput Sequencing of Human Hair
Source: PLoS One. 2011 Nov 16;6(11):e27603. doi: 10.1371/journal.pone.0027603 (PMC3218001; doi:10.1371/journal.pone.0027603)
Supplement: Methods S1 — Supplemental methods. (DOC) [file pone.0027603.s006.doc]

**Methods S1**

**Demographic profile of participants**

Parietal and vertex hair from five de-identified individuals were used for the hair shaft

RNA library. The demographic profile of collected hair included two males, three

females, and aged 21 to 40 years old (average 31.6). Populations of origin included

India, Korea, USA, and China. Hair morphologies included three individuals with straight

hair and two individuals with wavy hair.

**RNA extraction from hair**

For RNA extraction from a highly crosslinked, keratinized tissue, we modified a Gough

RNA extraction buffer (7 M urea, 1% SDS, 0.35M NaCl, 10 mM EDTA, 10 mM Tris-HCl)

[47] by the addition of a reducing agent, 100 mM DTT. RNA extraction was accelerated

by mechanical bead disruption of the hair (1.0 mm zirconia beads; BioSpec Products,

Inc.). Following lysis, RNA was extracted with Trizol reagent (Life Technologies)

following manufacturer’s instructions and precipitated in isopropanol with sodium acetate

and glycogen. The pellet was washed in 70% ethanol, resuspended in DNase I buffer,

treated with RNase-free DNase (New England Biolabs), and inactivated with 10 mM

EDTA and heat. The sample was then precipitated again in ethanol, washed with 70%

ethanol, and resuspended in water. RNA concentration and purity was determined by

A260/280 absorbance using a Nanodrop ND-2000 Spectrophotometer (Thermo Fisher)

and Qubit fluorometer (Life technologies).

**Small RNA library preparation from hair**

Briefly, 1 μg total RNA is ligated onto 5´-riboadenylated-3´-DNA adapter with 3 units of

T4 RNA Ligase 2 (truncated) (New England Biolabs) in 1X T4RNL2 truncated Reaction

Buffer with 8 mM MgCl2 and 20 units RNase OUT™ and incubated for 20ºC for 1 hour,

followed by ligation to an unmodified RNA adapter with T4 RNA Ligase. This product is

then primed with the Illumina RT primer and reverse transcribed with Superscript II and

the cDNA product is amplified in 18 cycles of PCR. PCR product is purified on 10% TBE

PAGE gel and excised and eluted from the gel using standard crush and soak

procedures, and finally ethanol precipitated using glycogen as carrier reagent.

Sequencing was carried out using an Illumina GAIIx system.

**Sequence Analysis**

The raw RNA-seq data was generated in FASTQ format [2] by the Genome Analyzer

Pipeline, using a Genome Analyzer II from Illumina; the quality scores were offset by 64

following Solexa-1.3+ standards. Preprocessing of the FASTQ data was done with the

FASTX-Toolkit package. Adaptor trimming was done with the program 'fastx_clipper',

requiring a minimum adaptor alignment of 5 bases. The reads were quality filtered using

the command 'fastq_quality_filter', requiring that a minimum of 90% of the bases have a

quality score of 20 or higher. Following preprocessing, the library was aligned to the

genome using Bowtie software package [3]. The bowtie index was built using the

program 'bowtie-build', based upon the NBCI36/HG18 human genome, downloaded

from the UCSC Genome Browser [4]; non-canonical and unscaffolded chromosomes

were excluded. The library was aligned to the index using the command 'bowtie', with

the following parameters: seed length of 29 base pairs and seed mismatch at most 2,

giving up to 10 alignments, and excluding reads aligning >10 times. This was done on

the Triton Resource at the San Diego Supercomputer Center at UCSD; using a single

node of 32 processors and 512GB of RAM, each alignment took approximately 15

minutes. The alignment generated a SAM file, which was further processed using

Samtools [5]. The commands 'samtools view' and 'samtools sort' were used to generate

a BAM file of sorted alignments to the genome. This was converted into a BED file using

the BEDTools [6] command 'bamToBed', and per-gene and per-exon alignment counts

were generated using the command 'intersectBed'. For coding genes, expression counts

from 'intersectBed' were calculated in reference to the UCSC Genome Browser table

'refFlat', which includes all annotated regions in the REFSEQ release NCBI36 [7].

miRNA and snRNA counts were generated differently, using separate Bowtie genome

indexes built from the miRBase Release 16 [8] and snoRNAbase Version 3 [9]

databases, respectively. For miRBase, alignments to both pre-miRNA and mature

sequences were counted. For both miRBase and snoRNAbase the reference sequences

were artificially extended with 20 adenoside nucleotides on both ends, to allow for

alignement to alternatively spliced small RNAs. In accordance with the extremely high

target-specificity of miRNAs and snoRNAs, the following 'bowtie' parameters were used:

a seed length of 23 bases for miRBase, 29 for snoRNAdb, and a seed mismatch of 0; all

alignments were counted. Post-processing using Samtools and BEDTools was identical

to that done for the genome.
