# Supplementary material for: The Post-Apoptotic Fate of RNAs Identified Through High-Throughput Sequencing of Human Hair
Source: PLoS One. 2011 Nov 16;6(11):e27603. doi: 10.1371/journal.pone.0027603 (PMC3218001; doi:10.1371/journal.pone.0027603)
Supplement: References S1 — Supplemental references. (DOC) [file pone.0027603.s007.doc]

**Supplemental References**

Cock, P. J. A., Fields, C. J., Goto, N., Heuer, M. L., and Rice, P. M. (2010) The Sanger FASTQ file format for sequences with quality scores, and the Solexa/Illumina FASTQ variants. Nucleic Acids Res *38*, 1767-1771.

Fujita, P. A., Rhead, B., Zweig, A. S., Hinrichs, A. S., Karolchik, D., Cline, M. S., Goldman, M., Barber, G. P., Clawson, H., Coelho, A., et al. (2011) The UCSC Genome Browser database: update 2011. Nucleic Acids Res *39*, D876-882.

Kozomara, A., and Griffiths-Jones, S. (2011) miRBase: integrating microRNA annotation and deep-sequencing data. Nucleic Acids Res *39*, D152-157.

Lander, E. S., Linton, L. M., Birren, B., Nusbaum, C., Zody, M. C., Baldwin, J., Devon, K., Dewar, K., Doyle, M., FitzHugh, W., et al. (2001). Initial sequencing and analysis of the human genome. Nature *409*, 860-921.

Langmead, B., Trapnell, C., Pop, M., and Salzberg, S. L. (2009) Ultrafast and memory-efficient alignment of short DNA sequences to the human genome. Genome Biol *10*, R25.

Lestrade, L., and Weber, M. J. (2006) snoRNA-LBME-db, a comprehensive database of human H/ACA and C/D box snoRNAs. Nucleic Acids Res *34*, D158-162.

Li, H., Handsaker, B., Wysoker, A., Fennell, T., Ruan, J., Homer, N., Marth, G., Abecasis, G., and Durbin, R. (2009) The Sequence Alignment/Map format and SAMtools. Bioinformatics *25*, 2078-2079.

Quinlan, A. R., and Hall, I. M. (2010) BEDTools: a flexible suite of utilities for comparing genomic features. Bioinformatics *26*, 841-842.
